# A New Fuzzy System Based on Rectangular Pyramid

**DOI:** 10.1155/2015/682989

**Published:** 2015-03-19

**Authors:** Mingzuo Jiang, Xuehai Yuan, Hongxing Li, Jiaxia Wang

**Affiliations:** ^1^Faculty of Electronic Information and Electrical Engineering, Dalian University of Technology, Dalian 116024, China; ^2^Foundation Department, Dalian University of Technology, Panjin 124221, China; ^3^Faculty of Vehicle Engineering and Mechanics, Dalian University of Technology, Dalian 116024, China

## Abstract

A new fuzzy system is proposed in this paper. The novelty of the proposed system is mainly in the compound of the antecedents, which is based on the proposed rectangular pyramid membership function instead of t-norm. It is proved that the system is capable of approximating any continuous function of two variables to arbitrary degree on a compact domain. Moreover, this paper provides one sufficient condition of approximating function so that the new fuzzy system can approximate any continuous function of two variables with bounded partial derivatives. Finally, simulation examples are given to show how the proposed fuzzy system can be effectively used for function approximation.

## 1. Introduction

Fuzzy system has been the subject of numerous researches in more than three decades. It has been successfully applied to a great variety of different processes such as control engineering, signal processing, information processing, machine intelligence, decision making, management, finance, medicine, and robotics [1–9].

Motivated by successful applications of fuzzy system, there have been a number of works aiming at improving the structure and performance of fuzzy system. Yuan et al. [10] put forward a parameter singleton fuzzifier method. Celikyilmaz and Turksen [11] presented a new fuzzy system modeling approach based on improved fuzzy functions to model systems with continuous output variable. Adaptive fuzzy systems are also developed well. In [12], a dynamic rule base which allows the fuzzy sets to dynamically change or move with the inputs was used for the construction of fuzzy system. Márquez et al. [13] proposed adaptive t-norms for the antecedents connection and adaptive defuzzification methods. Moreover, fuzzy system can be integrated with some other techniques, for example, neurocontrol [14–17], genetic algorithms and particle swarm optimization [18–23], and fuzzy sliding-mode control [24–26]. This paper focuses on the structure design of conventional dual-input single-output Mamdani fuzzy system to improve its approximation property and simplify its structure. A new kind of fuzzy system based on the proposed rectangular pyramid membership function is established. The model of the rectangular pyramid fuzzy system (RPFS) is introduced mainly by replacing the compound of the two rule antecedents using t-norm with the rectangular pyramid membership function of the input vector. With the help of rectangular pyramid membership function, the fuzzy system structure becomes simple and easily realized.

In most applications of fuzzy systems, the main design objective can be considered as problems of functions approximation. So the study on approximation theory of fuzzy systems is very important and necessary. Wang [27] used Stone-Weierstrass Theorem to prove the approximation capability of a common kind of fuzzy systems. Based on the above research, Wang and Mendel [28] proposed fuzzy basic functions to explain the approximation property of fuzzy systems. In [29], the approximation properties of MIMO fuzzy systems are discussed based on its fuzzy basic functions. Castro [30] proved the approximation properties of the fuzzy systems with a wide class of fuzzy logics and membership functions. Mao et al. [31] addressed whether a fuzzy system with weaker constraints to its membership functions can be a universal approximator. Li [32] found out that the commonly used fuzzy system algorithms can be regarded as some interpolation functions. Generally speaking, fuzzy systems can approximate any continuous function on any compact domain, which explains the ability of fuzzy controller in achieving satisfactory performance in applications. So before the application of one kind of fuzzy systems, it is helpful to know clearly whether they are universal approximators. Note that the features of RPFS are mainly determined by the properties of its fuzzy basic functions. We will first give an analysis of the properties of fuzzy basic functions of RPFS and then discuss the approximation property of RPFS.

Sufficient conditions of fuzzy systems lead to the following practical result: the derived formulas can calculate the numbers of input fuzzy sets and fuzzy rules needed to satisfy any given approximation accuracy. Ying [33] gave sufficient conditions for general fuzzy system. Chen [34] established sufficient conditions for two classes of fuzzy logic controllers in [33]. In [35], the sufficient conditions for Boolean fuzzy systems were proposed by using Weierstrass Theorem. Zeng et al. [36] and Liu et al. [37] made a systematic and comparative study on sufficient conditions for different fuzzy systems. In a constructive way, we have found one sufficient condition on the premise that RPFS can uniformly approximate any real continuous function on a compact domain to any degree of accuracy.

The structure of this paper is as follows. After the introduction, the model of the conventional fuzzy system and its approximation theory are given in Section 2. The construction of RPFS is introduced in Section 3. Some definitions and properties of RPFS are given in Section 4 in order to describe the approximation capability of RPFS. One sufficient condition of RPFS is given in Section 5. After the above discussion, we provide some simulation results of approximating functions to evaluate the approximation performance of RPFS in Section 6. Conclusions are made in the last section.

## 2. Preliminaries

In this section, we will introduce the model of conventional dual-input single-output fuzzy system. Furthermore, we will describe the ability of the system in approximating any continuous function on an arbitrary compact domain.

Conventional fuzzy system consists of four principal components: fuzzifier, fuzzy rule base, fuzzy inference engine, and defuzzifier. The fuzzy rule base contains information of how to infer new control actions. The fuzzy inference engine is a reasoning mechanism which performs inference procedure on the fuzzy rules and derives reasonable control actions. It is the central part of a fuzzy system. The fuzzification interface (or fuzzifier) defines a mapping from a real-valued space to a fuzzy space, and the defuzzification interface (or defuzzifier) defines a mapping from a fuzzy space to a real-valued space. The fuzzifier converts a crisp value to a fuzzy number while the defuzzifier converts the inferred fuzzy conclusion to a crisp value.

Consider a dual-input single-output fuzzy system: *U* × *V* → *W*, where *U* × *V* ⊂ *R* ^2^ is the input space and *W* ⊂ *R* is the output space, respectively. The fuzzy rule base consists of *MN* rules in the following form:

(1) $$\begin{array}{c} R_{ij}:\text{IF}\;x\;\text{is}\;A_{i}^{1},\;\;y\;\text{is}\;A_{j}^{2},\;\;\text{THEN}\;z\;\text{is}\;B_{ij}, \end{array}$$

where *i* = 1,2,…, *M*; *j* = 1,2,…, *N*; *x* and *y* are the input variables of the fuzzy system; *z* is the output variable of the fuzzy system. *A*_*i*_^1^ ⊂ *U*, *A*_*j*_^2^ ⊂ *V*, and *B*_*ij*_ ⊂ *W* are linguistic terms characterized by fuzzy membership functions *A*_*i*_^1^(*x*), *A*_*j*_^2^(*y*), and *B*_*ij*_(*z*), respectively. *z*_*ij*_ is the point in *W* at which *B*_*ij*_(*z*) achieves its maximum value. Assume that the fuzzifier is the singleton fuzzifier method. Under the commonly used fuzzy engine and defuzzifier method, the final output of the conventional dual-input single-output fuzzy system is derived as follows:

(2) $$\begin{array}{c} z=\frac{\sum_{i=1}^{M}\sum_{j=1}^{N}A_{i}^{1}\left(x\right)A_{j}^{2}\left(y\right)z_{ij}}{\sum_{i=1}^{M}\sum_{j=1}^{N}A_{i}^{1}\left(x\right)A_{j}^{2}\left(y\right)}. \end{array}$$

The below theorem [27] gives the basic approximation property of conventional dual-input single-output fuzzy system.

**Theorem 1 .**

For any given real continuous function *g*(*x*, *y*) on the compact set *U* × *V* ⊂ *R* ^2^ and arbitrary *ε* > 0, there exists a fuzzy system *f*(*x*, *y*) such that

(3) $$\begin{array}{c} \underset{(x,y)\in U\times V}{\sup}\left|g\left(x,y\right)-f\left(x,y\right)\right|<\varepsilon . \end{array}$$

## 3. Construction of Rectangular Pyramid Fuzzy System

**Figure 1:**
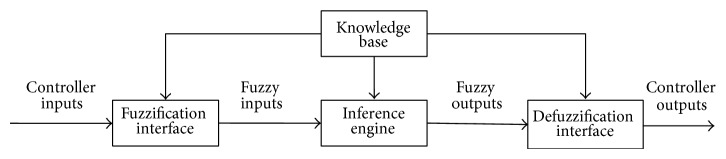
The structure of RPFS.

**Figure 2:**
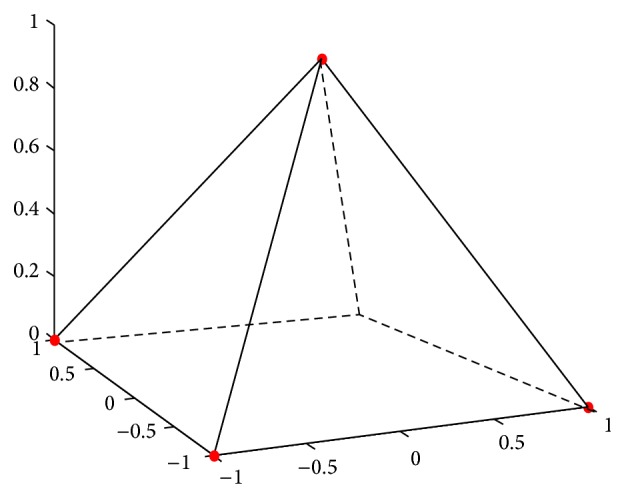
Rectangular pyramid membership function.

In this section, we applied the definition of rectangular pyramid membership function instead of t-norm to the construction of fuzzy system to achieve high accuracy of the output of given data. Moreover, the structure of fuzzy system is simplified. A detailed description of RPFS is given. The discussion is limited to dual-input single-output systems. RPFS consists of four principle parts which are similar to those of conventional fuzzy systems: fuzzifier, fuzzy rule base, fuzzy inference engine, and defuzzifier. For this class of fuzzy systems, they can be constructed by Sections 3.1–3.3. The structure of RPFS is presented in Figure 1 and the sketch of rectangular pyramid membership function is shown in Figure 2.

### 3.1. Fuzzifier

The fuzzification interface can translate input values into linguistic terms which are characterized by the rectangular pyramid membership functions. Denote by (*x*, *y*) the input vector and by *z* the output variable. Let [*a*, *b*] × [*c*, *d*] be the universe of the input vector and [*m*, *n*] the universe of the output variable. The universe [*a*, *b*] × [*c*, *d*] is equidistantly divided by peak points (*x*_*i*_, *y*_*j*_)  (*i* = 1,2,…, *M*; *j* = 1,2,…, *N*) of the rectangular pyramid membership functions. Consider one subset [*x*_*i*_, *x*_*i*+1_]×[*y*_*j*_, *y*_*j*+1_] in [*a*, *b*] × [*c*, *d*]; for simplicity of discussion, we construct, respectively, the rectangular pyramid membership functions *P*_*ij*_(*x*, *y*), *P*_*i*.*j*+1_(*x*, *y*), *P*_*i*+1,*j*+1_(*x*, *y*), and *P*_*i*+1,*j*_(*x*, *y*) (on the small area [*x*_*i*_, *x*_*i*+1_]×[*y*_*j*_, *y*_*j*+1_]) of the peak points (*x*_*i*_, *y*_*j*_), (*x*_*i*_, *y*_*j*+1_), (*x*_*i*+1_, *y*_*j*+1_), and (*x*_*i*+1_, *y*_*j*_) as follows.

At point (*x*_*i*_, *y*_*j*_), when *x*_*i*_ ≤ *x* ≤ *x*_*i*+1_, *y*_*j*_ ≤ *y* ≤ *y*_*j*_ + ((*y*_*j*+1_ − *y*_*j*_)/(*x*_*i*+1_ − *x*_*i*_))(*x* − *x*_*i*_), we have

(4) $$\begin{array}{c} P_{ij}\left(x,y\right)=\frac{x_{i+1}-x}{x_{i+1}-x_{i}}; \end{array}$$

when *x*_*i*_ ≤ *x* ≤ *x*_*i*+1_, *y*_*j*_ + ((*y*_*j*+1_ − *y*_*j*_)/(*x*_*i*+1_ − *x*_*i*_))(*x* − *x*_*i*_) ≤ *y* ≤ *y*_*j*+1_, we have

(5) $$\begin{array}{c} P_{ij}\left(x,y\right)=\frac{y_{j+1}-y}{y_{j+1}-y_{j}}. \end{array}$$

At point (*x*_*i*_, *y*_*j*+1_), when *x*_*i*_ ≤ *x* ≤ *x*_*i*+1_, *y*_*j*_ ≤ *y* ≤ *y*_*j*+1_ + ((*y*_*j*_ − *y*_*j*+1_)/(*x*_*i*+1_ − *x*_*i*_))(*x* − *x*_*i*_), we have

(6) $$\begin{array}{c} P_{i,j+1}\left(x,y\right)=\frac{y-y_{j}}{y_{j+1}-y_{j}}; \end{array}$$

when *x*_*i*_ ≤ *x* ≤ *x*_*i*+1_, *y*_*j*+1_ + ((*y*_*j*_ − *y*_*j*+1_)/(*x*_*i*+1_ − *x*_*i*_))(*x* − *x*_*i*_) ≤ *y* ≤ *y*_*j*+1_, we have

(7) $$\begin{array}{c} P_{i,j+1}\left(x,y\right)=\frac{x_{i+1}-x}{x_{i+1}-x_{i}}. \end{array}$$

At point (*x*_*i*+1_, *y*_*j*+1_), when *x*_*i*_ ≤ *x* ≤ *x*_*i*+1_, *y*_*j*_ ≤ *y* ≤ *y*_*j*_ + ((*y*_*j*+1_ − *y*_*j*_)/(*x*_*i*+1_ − *x*_*i*_))(*x* − *x*_*i*_), we have

(8) $$\begin{array}{c} P_{i+1,j+1}\left(x,y\right)=\frac{y-y_{j}}{y_{j+1}-y_{j}}; \end{array}$$

when *x*_*i*_ ≤ *x* ≤ *x*_*i*+1_, *y*_*j*_ + ((*y*_*j*+1_ − *y*_*j*_)/(*x*_*i*+1_ − *x*_*i*_))(*x* − *x*_*i*_) ≤ *y* ≤ *y*_*j*+1_, we have

(9) $$\begin{array}{c} P_{i+1,j+1}\left(x,y\right)=\frac{x-x_{i}}{x_{i+1}-x_{i}}. \end{array}$$

At point (*x*_*i*+1_, *y*_*j*_), when *x*_*i*_ ≤ *x* ≤ *x*_*i*+1_, *y*_*j*_ ≤ *y* ≤ *y*_*j*+1_ + ((*y*_*j*_ − *y*_*j*+1_)/(*x*_*i*+1_ − *x*_*i*_))(*x* − *x*_*i*_), we have

(10) $$\begin{array}{c} P_{i+1,j}\left(x,y\right)=\frac{x-x_{i}}{x_{i+1}-x_{i}}; \end{array}$$

when *x*_*i*_ ≤ *x* ≤ *x*_*i*+1_, *y*_*j*+1_ + ((*y*_*j*_ − *y*_*j*+1_)/(*x*_*i*+1_ − *x*_*i*_))(*x* − *x*_*i*_) ≤ *y* ≤ *y*_*j*+1_, we have

(11) $$\begin{array}{c} P_{i+1,j}\left(x,y\right)=\frac{y_{j+1}-y}{y_{j+1}-y_{j}}. \end{array}$$

The singleton fuzzifier method is adopted in this step. It maps a real-valued point (*x*, *y*) ∈ [*a*, *b*] × [*c*, *d*] into a fuzzy singleton set *P* ^*^ which has membership value 1 at (*x*, *y*) and 0 at the other points in [*a*, *b*] × [*c*, *d*]; that is,

(12) $$\begin{array}{c} P^{*}\left(x,y\right)=\left\{\begin{array}{cc} 1, & \text{if}\;\left(x,y\right)=\left(x^{\prime },y^{\prime }\right)\\ 0, & \text{otherwise}. \end{array}\right. \end{array}$$

### 3.2. Fuzzy Inference

The fuzzy inference engine is a decision-making mechanism that employs fuzzy rules from the fuzzy rule base to determine a mapping from the fuzzy sets in the input space to the fuzzy sets in the output space.

The conventional fuzzy rule base consists of a set of linguistic rules in the form of (1). In this paper, we consider the fuzzy rules which are different from those of the conventional fuzzy system in the following form:

(13) Rij:IF  x,y  is  Pij,    THEN  z  is  Cij  i=1,2,…,M;j=1,2,…,N,

where *P*_*ij*_ are the fuzzy set characterized by rectangular pyramid membership function *P*_*ij*_(*x*, *y*). The difference between RPFS and the conventional fuzzy system mainly lies in the compound of the antecedents. In the conventional fuzzy system, fuzzy intersections (t-norms) for connective “and” are used. Then, the compound fuzzy proposition

(14) $$\begin{array}{c} \text{IF}\;x\;\text{is}\;A_{i}^{1},\;\;y\;\text{is}\;A_{j}^{2} \end{array}$$

is interpreted as a fuzzy set *A*_*i*_^1^∩*A*_*j*_^2^ in *U* × *V* with a membership function

(15) $$\begin{array}{c} \mu_{A_{i}^{1}\cap A_{j}^{2}}\left(x,y\right)=t\left[\mu_{A_{i}^{1}}\left(x\right),\mu_{A_{j}^{2}}\left(y\right)\right], \end{array}$$

where *t* : [0,1]×[0,1]→[0,1] is any t-norm.

As the fuzzy rule base consists of a set of rules, the relationship among these rules is an interesting question. Important properties of a set of conventional rules are completeness, consistency, and continuity. Note that the form of fuzzy rules in this paper has been changed; it is necessary to know whether the changed rules have the similar good character as the conventional rules.

**Definition 2 .**

A set of fuzzy IF-THEN rules is complete if, for any (*x*, *y*) ∈ *U* × *V*, there exists at least one rule in the fuzzy rule base, say rule *R*_st_ (in the form of (13)), such that

(16) $$\begin{array}{c} P_{\text{st}}\left(x,y\right)\neq 0\;\forall i=1,2,\ldots ,M;\;\;j=1,2,\ldots ,N. \end{array}$$

Intuitively, the completeness of a set of rules means that at any point in the input space there exists at least one rule making the membership value of the IF part of the rule at this point nonzero.

**Definition 3 .**

A set of fuzzy IF-THEN rules is consistent if there are no rules with the same IF parts but different THEN parts.

**Definition 4 .**

A set of fuzzy IF-THEN rules is continuous if there do not exist such neighboring rules whose THEN part fuzzy sets have empty intersection.

It is obvious that the rule base of RPFS has properties of completeness, consistency, and continuity.

For fuzzy rule base containing more than one rule, the key question is how to infer with a set of rules. There are two ways: composition-based inference and individual-rule based inference. If all the *MN* fuzzy rules are firstly composed into a new fuzzy rule which is then used to generate a fuzzy consequence in accordance with the given fuzzy antecedents, this is the so-called composition-based inference. Alternatively, each of the *MN* fuzzy rules is individually used to generate a fuzzy consequence in accordance with the given fuzzy antecedent. The resulting *MN* fuzzy consequences are then composed into a new fuzzy consequence. This is the so-called individual-rule based inference. In this paper, we follow the widely used composition-based inference. So each fuzzy rule can be written as

(17) $$\begin{array}{c} R_{ij}\left(x,y,z\right)=\theta \left(P_{ij}\left(x,y\right),C_{ij}\left(z\right)\right)=P_{ij}\left(x,y\right)C_{ij}\left(z\right), \end{array}$$

where *θ* is the Mamdani product implication operator from the fuzzy antecedent *P*_*ij*_ to the fuzzy consequence *C*_*ij*_. Based on composition-based inference, we have

(18) $$\begin{array}{c} R=\overset{M}{\underset{i=1}{⋃}}\overset{N}{\underset{j=1}{⋃}}R_{ij}. \end{array}$$

Therefore, the membership function of *R* can be expressed as

(19) $$\begin{array}{c} R\left(x,y,z\right)=\overset{M}{\underset{i=1}{⋁}}\overset{N}{\underset{j=1}{⋁}}\left[P_{ij}\left(x,y\right)C_{ij}\left(z\right)\right]. \end{array}$$

*R* composes the *MN* fuzzy rules into a single fuzzy rule via the union operator. To derive the expression of the output, we need to construct a set transformation *T* : *P* ^*^ → *C* ^*^, *C* ^*^ = [(*P* ^*^ × *Z*)∩*R*]_*z*_,

(20) C∗z=⋁x′,y′∈X×YP∗x′,y′∧Rx′,y′,z=Rx,y,z.

### 3.3. Defuzzifier

In this step, we need a defuzzification process to get a crisp decision. Among the commonly used defuzzification strategies, fuzzy centroid-defuzzification method yields superior results [38]. Through this method, we get an equation of output variable

(21) z=∫mnzC∗zdz∫mnC∗zdz=∫mnz⋁i=1M⋁j=1NPijx,yCijzdz∫mn⋁i=1M⋁j=1NPijx,yCijzdz≈∑t=1MNzt[⋁i=1M⋁j=1N(Pijx,yCijz)]Δzt∑t=1MN[⋁i=1M⋁j=1N(Pijx,yCijz)]Δzt=Pi,jx,yzi,j+Pi+1,jx,yzi+1,j  +Pi+1,j+1x,yzi+1,j+1+Pi,j+1x,yzi,j+1 ×Pi,jx,y+Pi+1,jx,y   +Pi+1,j+1x,y+Pi,j+1x,y−1,

where *z*_*i*_1_,*j*_1__ = *f*(*x*_*i*_1__, *y*_*j*_1__)  (*i*_1_ = *i*, *i* + 1; *j*_1_ = *j*, *j* + 1) and *f*(*x*, *y*) is a real-valued function defined on a domain *D*′ ⊂ *R* ^2^. From the expressions of the rectangular pyramid membership functions *P*_*ij*_(*x*, *y*), *P*_*i*,*j*+1_(*x*, *y*), *P*_*i*+1,*j*+1_(*x*, *y*), and *P*_*i*+1,*j*_(*x*, *y*) and the above formula, the general structure of RPFS can be represented as follows:

(i) when *x*_*i*_ ≤ *x* ≤ *x*_*i*+1_, *y*_*j*_ ≤ *y* ≤ *y*_*j*_ + ((*y*_*j*+1_ − *y*_*j*_)/(*x*_*i*+1_ − *x*_*i*_))(*x* − *x*_*i*_), *y*_*j*_ ≤ *y* ≤ *y*_*j*+1_+((*y*_*j*_ − *y*_*j*+1_)/(*x*_*i*+1_ − *x*_*i*_))(*x* − *x*_*i*_),
  (22) Sx,y=xi+1−xyj+1−yj(2y+yj+1−3yj)xi+1−xizij+y−yj2y+yj+1−3yjzi,j+1+y−yj2y+yj+1−3yjzi+1,j+1+x−xiyj+1−yj2y+yj+1−3yjxi+1−xizi+1,j;
(ii) when *x*_*i*_ ≤ *x* ≤ *x*_*i*+1_, *y*_*j*_ + ((*y*_*j*+1_ − *y*_*j*_)/(*x*_*i*+1_ − *x*_*i*_))(*x* − *x*_*i*_) ≤ *y* ≤ *y*_*j*+1_, *y*_*j*_ ≤ *y* ≤ *y*_*j*+1_+((*y*_*j*_ − *y*_*j*+1_)/(*x*_*i*+1_ − *x*_*i*_))(*x* − *x*_*i*_),
  (23) Sx,y=xi+1−xiyj+1−y2x+xi+1−3xiyj+1−yjzij+xi+1−xiy−yj2x+xi+1−3xiyj+1−yjzi,j+1+x−xi2x+xi+1−3xizi+1,j+1+x−xi2x+xi+1−3xizi+1,j;
(iii) when *x*_*i*_ ≤ *x* ≤ *x*_*i*+1_, *y*_*j*_ + ((*y*_*j*+1_ − *y*_*j*_)/(*x*_*i*+1_ − *x*_*i*_))(*x* − *x*_*i*_) ≤ *y* ≤ *y*_*j*+1_, *y*_*j*+1_ + ((*y*_*j*_ − *y*_*j*+1_)/(*x*_*i*+1_ − *x*_*i*_))(*x* − *x*_*i*_)≤*y* ≤ *y*_*j*+1_,
  (24) Sx,y=y−yj+1−2y+3yj+1−yjzij+yj+1−yjxi+1−x−2y+3yj+1−yjxi+1−xizi,j+1+yj+1−yjx−xi−2y+3yj+1−yjxi+1−xizi+1,j+1+yj+1−y−2y+3yj+1−yjzi+1,j;

otherwise

(25) Sx,y=xi+1−x−2x+3xi+1−xizij+xi+1−x−2x+3xi+1−xizi,j+1+xi+1−xiy−yj−2x+xi+1−xiyj+1−yjzi+1,j+1+xi+1−xiyj+1−y−2x+3xi+1−xiyj+1−yjzi+1,j.

**Figure 3:**
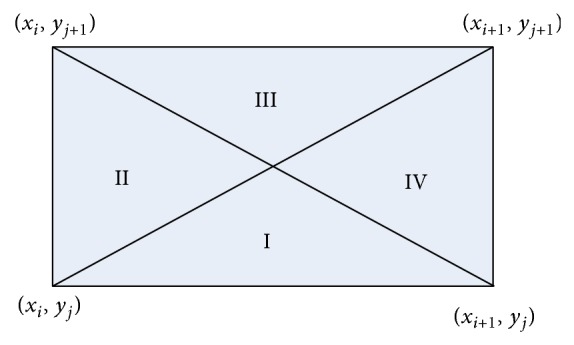
The division of small rectangular area [*x*_*i*_, *x*_*i*+1_]×[*y*_*j*_, *y*_*j*+1_] of *S*(*x*, *y*).

The four conditions correspond to the division domains I, II, III, and IV in Figure 3, respectively. In the rest of this paper, *S*(*x*, *y*) will be used to represent the output of RPFS and *P*_*ij*_(*x*, *y*) the rectangular pyramid membership function at peak point (*x*_*i*_, *y*_*j*_).

## 4. Approximation Property of Rectangular Pyramid Fuzzy System

In this section, in order to introduce the basic approximation property of RPFS, the definition and properties of fuzzy basic functions are firstly given. From the expressions of the output of RPFS, it is easy to find that *S*(*x*, *y*) can be represented by a linear combination of one kind of functions; for example,

(26) φi,jx,y=Pi,jx,yPi,jx,y+Pi+1,jx,y+Pi+1,j+1x,y+Pi,j+1x,y.

Thus, this kind of functions can be defined as fuzzy basic functions of RPFS. The exact definition is as follows.

**Definition 5 .**

Define fuzzy basic functions of RPFS as

(27) φi2,j2x,y=Pi2,j2x,yPi,jx,y+Pi+1,jx,y+Pi+1,j+1x,y+Pi,j+1x,y,

where *i*_2_ = *i*, *i* + 1; *j*_2_ = *j*, *j* + 1. According to Definition 5, it is obvious that

(28) φi,jx,y+φi+1,jx,y+φi+1,j+1x,y+φi,j+1x,y=1.

Now the output of RPFS can be represented as

(29) Sx,y=φi,jx,yzi,j+φi+1,jx,yzi+1,j+φi+1,j+1x,yzi+1,j+1+φi,j+1x,yzi,j+1,

where *φ*_*i*_2_,*j*_2__(*x*_*i*_3__, *y*_*j*_3__) = 1(*i*_3_ = *i*, *i* + 1; *j*_3_ = *j*, *j* + 1), when *i*_2_ = *i*_3_ and *j*_2_ = *j*_3_, and, inversely, *φ*_*i*_2_,*j*_2__(*x*_*i*_3__, *y*_*j*_3__) = 0, when *i*_2_ ≠ *i*_3_ or *j*_2_ ≠ *j*_3_.

From the above analysis, we can conclude as follows. The basic idea of RPFS is a kind of piecewise interpolation function with the conditions

(30) $$\begin{array}{c} S\left(x_{i},y_{j}\right)=f\left(x_{i},y_{j}\right)\;\left(i=1,2,\ldots ,M;j=1,2,\ldots ,N\right). \end{array}$$

Fuzzy basic functions play the same role in RPFS as the interpolation basis functions do in computational mathematics. The output of RPFS is a weighted sum with the corresponding fuzzy basic function values as the weights on [*x*_*i*_, *x*_*i*+1_] × [*y*_*j*_, *y*_*j*+1_].

Denote by *C*(*D*′) the collection of all the continuous functions mapping *D*′ into the real numbers. The distance between *f* and *g* in *C*(*D*′) can be measured as ‖*f* − *g*‖ = sup⁡_(*x*, *y*)∈*D*′_|*f*(*x*, *y*) − *g*(*x*, *y*)|. The problem of approximation can be described as follows: given *f*(*x*, *y*) ∈ *C*(*D*′) and any *ε* > 0, is it possible for RPFS to approximate the function *f*(*x*, *y*) on an arbitrary compact domain *D*′ to *ε* level? The following theorem addresses the above posed problem.

**Theorem 6 .**

For any given real continuous function *f*(*x*, *y*) on the compact set *D*′ ⊂ *R* ^2^ and arbitrary *ε* > 0, there exists a RPFS *S*(*x*, *y*) such that

(31) $$\begin{array}{c} \left\|S-f\right\|=\underset{\left(x,y\right)\in D^{\prime }}{\sup}\left|S\left(x,y\right)-f\left(x,y\right)\right|<\varepsilon . \end{array}$$

**Proof**

Without losing generality, the proof is discussed on one partition [*x*_*i*_, *x*_*i*+1_]×[*y*_*j*_, *y*_*j*+1_] of the domain *D*′. We can now prove the theorem with the help of the interpolation property of RPFS. From (30), we have

(32) Sx,y−fx,y =Sx,y−Sxi,yj+fxi,yj−fx,y ≤Sx,y−Sxi,yj+fxi,yj−fx,y.

Using Lagrange Mean Value Theorem, there exist (*ξ*_1_, *η*_1_) and (*ξ*_2_, *η*_2_) which both belong to (*x*_*i*_, *x*_*i*+1_)×(*y*_*j*_, *y*_*j*+1_) such that

(33) Sx,y−fx,y=∂S∂x(ξ1,η1)x−xi+∂S∂y(ξ1,η1)y−yj +∂f∂x(ξ2,η2)x−xi+∂f∂y(ξ2,η2)(y−yj)≤∂S∂xξ1,η1+∂S∂yξ1,η1+∂f∂xξ2,η2+∂f∂yξ2,η2h,

where *h* = max⁡⁡{|*x* − *x*_*i*_ | , |*y* − *y*_*j*_|}. As (|(∂*S*/∂*x*)|_(*ξ*_1_, *η*_1_)_ | +|(∂*S*/∂*y*)|_(*ξ*_1_, *η*_1_)_ | +|(∂*f*/∂*x*)|_(*ξ*_2_, *η*_2_)_| + |(∂*f*/∂*y*)|_(*ξ*_2_, *η*_2_)_|) is a constant, when *h* is sufficiently small, it is evident that for any *ε* > 0 the following inequality can be obtained:

(34) $$\begin{array}{c} \left|S\left(x,y\right)-f\left(x,y\right)\right|<\varepsilon \;\forall \left(x,y\right)\in D^{\prime }. \end{array}$$

Then

(35) $$\begin{array}{c} \left\|S-f\right\|=\underset{\left(x,y\right)\in D^{\prime }}{\sup}\left|S\left(x,y\right)-f\left(x,y\right)\right|<\varepsilon . \end{array}$$

The proof is completed.

In conclusion, Theorem 6 shows that RPFS is capable of approximating any real continuous function of two variables on a compact set to arbitrary accuracy.

## 5. Sufficient Condition of Approximation of Rectangular Pyramid Fuzzy System

In this section, we will establish one sufficient condition on the premise that RPFS can approximate any continuous functions of two variables on a compact domain (in Section 4). It is impossible to give a formula of the needed rule number of RPFS to satisfy the required approximation accuracy for all continuous functions. However, for a special class of continuous functions, this is possible. In the below theorem, continuous functions with bounded partial derivatives are approximated by RPFS. Now, we present the main result of this section.

**Theorem 7 .**

Let *f* : [*a*, *b*] × [*c*, *d*] → [*m*, *n*] be a continuous function which satisfies *f*_*x*_(*x*, *y*) ≤ *M*_1_ and *f*_*y*_(*x*, *y*) ≤ *M*_2_, where *M*_1_ and *M*_2_ are both constants. For any approximation error bound *ε* > 0, there exists a RPFS *S*(*x*, *y*) that satisfies |*S*(*x*, *y*) − *f*(*x*, *y*)| < *ε*(∀(*x*, *y*) ∈ [*a*, *b*] × [*c*, *d*]) when

(36) $$\begin{array}{c} N_{1}=\max\left(N,M\right)>\frac{4M_{1}\left(b-a\right)+4M_{2}\left(d-c\right)}{\varepsilon }. \end{array}$$

**Proof**

Note that, from (28), we have

(37) Sx,y−fx,y =φijx,yfij+φi+1,jx,yfi+1,j   +φi+1,j+1x,yfi+1,j+1   +φi,j+1x,yfi,j+1−fx,y =φijx,yfij−fx,y     +φi+1,jx,yfi+1,j−fx,y   +φi+1,j+1x,yfi+1,j+1−fx,y   +φi,j+1x,yfi,j+1−fx,y.

Applying the triangle inequality and Lagrange Mean Value Theorem, we get, in light of *φ*_*i*_1_,*j*_1__(*x*, *y*) ≤ 1  (*i*_1_ = *i*, *i* + 1; *j*_1_ = *j*, *j* + 1),

(38) Sx,y−fx,y≤fij−fx,y+fi+1,j−fx,y +fi+1,j+1−fx,y+fi,j+1−fx,y=fxξ1′,η1′x−xi+fyξ1′,η1′y−yj +fxξ2′,η2′x−xi+1+fyξ2′,η2′y−yj +fxξ3′,η3′x−xi+1+fyξ3′,η3′y−yj+1 +fxξ4′,η4′x−xi+fyξ4′,η4′y−yj+1≤M1x−xi+M2y−yj +M1x−xi+1+M2y−yj +M1x−xi+1+M2y−yj+1 +M1x−xi+M2y−yj+1.

It is simple to show that

(39) $$\begin{array}{c} \left|x-x_{i}\right|\leq \frac{b-a}{M},\;\;\left|x-x_{i+1}\right|\leq \frac{b-a}{M},\\ \left|y-y_{j}\right|\leq \frac{d-c}{N},\;\;\left|y-y_{j+1}\right|\leq \frac{d-c}{N}. \end{array}$$

So let

(40) $$\begin{array}{c} \left|S\left(x,y\right)-f\left(x,y\right)\right|\leq 4M_{1}\frac{b-a}{M}+4M_{2}\frac{d-c}{N}<\varepsilon , \end{array}$$

Where (*ξ*_1_′, *η*_1_′), (*ξ*_2_′, *η*_2_′), (*ξ*_3_′, *η*_3_′), and (*ξ*_4_′, *η*_4_′)∈(*x*_*i*_, *y*_*j*_)×(*x*_*i*+1_, *y*_*j*+1_). Simplifying the above formula, we obtain

(41) $$\begin{array}{c} N_{1}=\max\left(N,M\right)>\frac{4M_{1}\left(b-a\right)+4M_{2}\left(d-c\right)}{\varepsilon }. \end{array}$$

The proof is completed.

## 6. Simulation

In this section, we compare the approximation performance of RPFS with the conventional fuzzy system (using Gaussian membership functions) mentioned in Section 2 by approximating four typical functions as follows:

(42) f1x,y=0.52+0.1x3+0.28y3−0.06xy,iiiiiiiix,y∈−1,1×−1,1;f2(x,y)=x3+y3, x,y∈[−1,1]×[−1,1];f3(x,y)=sin(x+y), x,y∈[−1,1]×[−1,1];f4(x,y)=0.5sin(xy), x,y∈[−1,1]×[−1,1].

**Figure 4:**
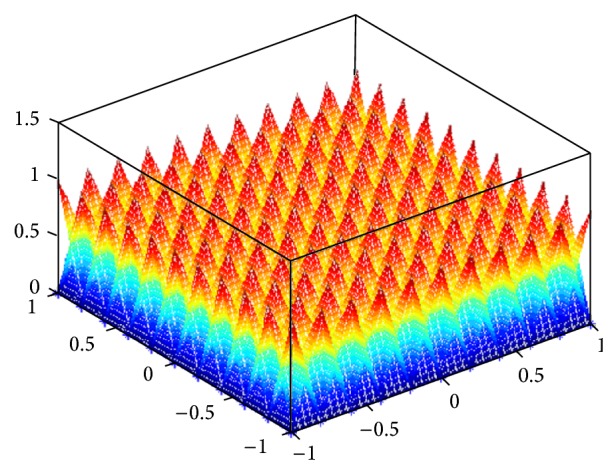
Membership functions of rule antecedents of RPFS in the simulation.

**Figure 5:**
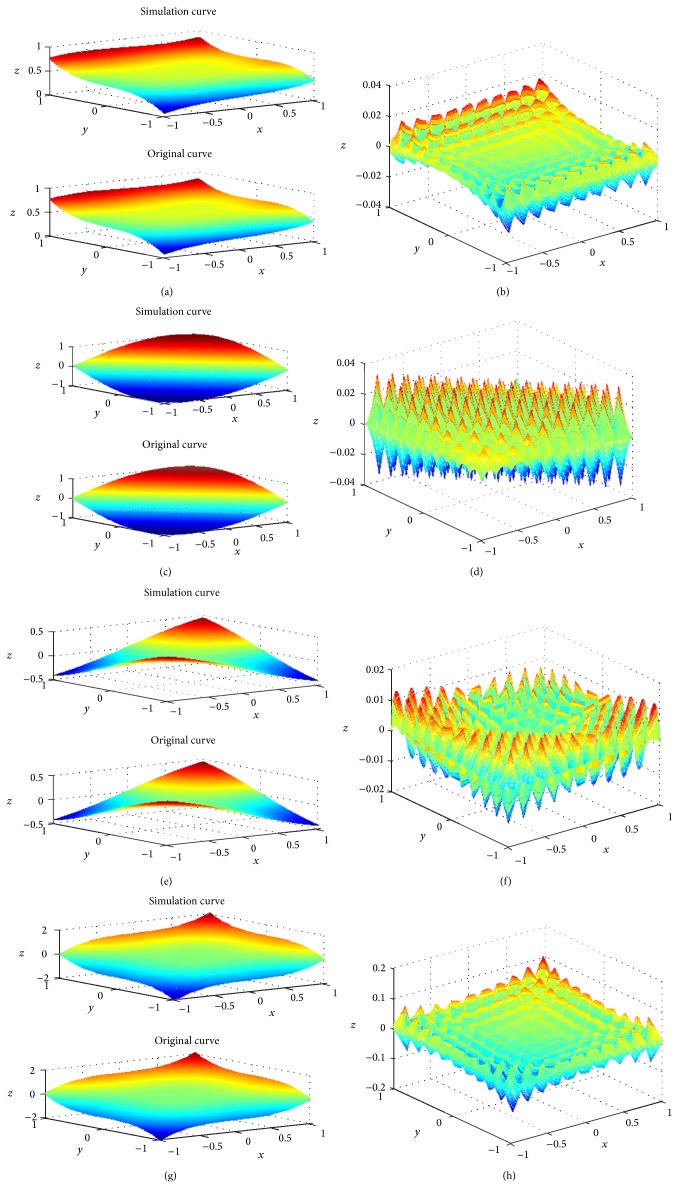
(a) The original and simulation surfaces of *f*_1_. (b) The approximation error surface of *f*_1_. (c) The original and simulation surfaces of *f*_2_. (d) The approximation error surface of *f*_2_. (e) The original and simulation surfaces of *f*_3_. (f) The approximation error surface of *f*_3_. (g) The original and simulation surfaces of *f*_4_. (h) The approximation error surface of *f*_4_.

For convenience, some notations are stated as follows: System I represents rectangular pyramid fuzzy system and System II represents the conventional fuzzy system (mentioned in Section 2) using Gaussian membership functions. Let the distances between the peak points of the two fuzzy systems be 0.2, and the distances between the sample points are chosen as 0.01. The membership functions of rule antecedents of RPFS are given in Figure 4. The original and simulation surfaces and the approximation error surfaces are shown in Figure 5. The max approximation errors and the standard deviations of System I and System II are presented in Tables 1 and 2, respectively.

It can be seen from Figure 5 that the simulation surfaces of RPFS almost coincided with the original surfaces. RPFS achieves a better approximation performance comparing with the conventional fuzzy system as shown in Tables 1 and 2. We can conclude that the proposed fuzzy system improves the approximation capability of the conventional fuzzy system to some extent.

## 7. Conclusions

A new type of fuzzy system based on the rectangular pyramid membership function is proposed in this paper. The model of the system has been introduced mainly by replacing the compound of the two rule antecedents using t-norm with the rectangular pyramid membership function, and the concrete derivation process is given. As the application problem of fuzzy system is essentially a function approximation problem, it is necessary to know about the approximation capability of RPFS. We proved that RPFS can approximate any continuous function of two variables to arbitrary degree of accuracy on any compact domain. Furthermore, the sufficient condition which reflects the relationship between the rule number and the approximation accuracy is also given combing the approximation property of RPFS. Finally, the simulation results demonstrate that the approximation performance of RPFS is found to be better than the conventional fuzzy system in the maximum approximation errors and the standard deviations. It means that RPFS has successfully improved the performance of function approximation of the conventional fuzzy system to some degree.

## Figures and Tables

**Table 1:** The maximum approximation errors of System I and System II.

Function	Error	
	System I	System II
*f*_1_	0.0212	0.0344
*f*_2_	0.1211	0.2004
*f*_3_	0.0353	0.0638
*f*_4_	0.0217	0.0214

**Table 2:** The standard deviations of System I and System II.

Function	Standard deviation	
	System I	System II
*f*_1_	0.0056	0.0097
*f*_2_	0.0265	0.0457
*f*_3_	0.0126	0.0247
*f*_4_	0.0040	0.0083
